# Association of *staphylococcus cohnii subspecies urealyticum* infection with recurrence of renal staghorn stone

**Published:** 2015

**Authors:** Zahra Shahandeh, Hamid Shafi, Farahnaz Sadighian

**Affiliations:** 1 Paramedical Faculty, Babol University of Medical Sciences, Iran.; 2 Department of Surgery, Shahid Beheshti Hospital, Babol University of Medical Sciences, Iran.

**Keywords:** *Staphylococcus cohnii subspecies urealyticum*, *Renal staghhorn stone*, *UTI*

## Abstract

**Background:**

*Stphylococcus cohnii* is an organism of coagulase negative species which is considered as normal flora. However, it has been isolated from urinary tract infections and surgical prostheses but its relation with staghorn stones has not been reported, yet.

**Case Presentation:**

A 50-years-old woman presented with left renal staghorn stone in June 2014. She had bilateral staghorn stones 7 years ago*. Staphylococcus cohnii subspecies urealyticum* were detected from a removed stone. After 7 years, recurrence staghorn stone in her left kidney was diagnosed and patient underwent another surgery. The patient had several attacks of cystitis during these 7 years. The results of stone and urine cultures revealed *staphylococcus cohnii subspecies urealyticum.*

**Conclusion:**

This case report emphasizes a possible association between *staphylococcus cohnii subspecies urealyticum* infection and recurrence renal staghhorn stone.

*Staphylococcus cohnii,* subspecies *urealyticum *(*S.cohnii sub.urealyticum*) is negative-coagulase staphylococci (NCS) and as a part of normal flora on the skin (1, 2). This bacterium can cause infection in immunocompromised patients as an opportunistic pathogen. Although the infection through this bacterium is uncommon, it has been reported in bacteremia associated with catheters and urinary tract infection (3). Stone disease is the most common problems in modern society and its probability appearing in the kidney is estimated 1% to 15% over the life. Today, it is proven that the bacteria which are urease-positive can form struvite stones. This type of stone is more common in people who are frequently suffering from UTI. The most common organisms causing infectious nephroliths have been *staphylococcus* and *Proteus mirabilis *(4). According to the investigations, the isolation of S.cohnii* sub.urealyticum* from the staghorn nephrolith has not been reported, yet. This study reports a possible role of *S.cohnii sub.urealyticum* infection in recurrence renal staghorn stone. 

## Case Presentation:

A 50-years-old woman presented with left renal staghorn stone in June 2014. She had a history of bilateral staghorn stones which was removed by anatrophic nephrolithotomy 7 years ago. At the first operation, the results of the removed stone culture revealed *S.cohnii sub.urealyticum* whereas, bladder urine culture was negative. After one year, there was no evidence of renal stone by ultrasonography examination.

However, the patient had several attacks of cystitis which was treated symptomatically without urinary examination. The patient did not adhere to further investigations for 7 years. A recurrence staghorn stone in the left kidney was confirmed by complete radiographic examination (picture1) and the patient underwent another surgery. At the second anatrophic nephrolithotomy operation, *S.cohnii sub.urealyticum* was isolated from stone and urine cultures, again. Chemical analysis showed that the stone was combined with magnesium- ammonium-phosphate and triple phosphate (staghorn stone). 

**Figure:**
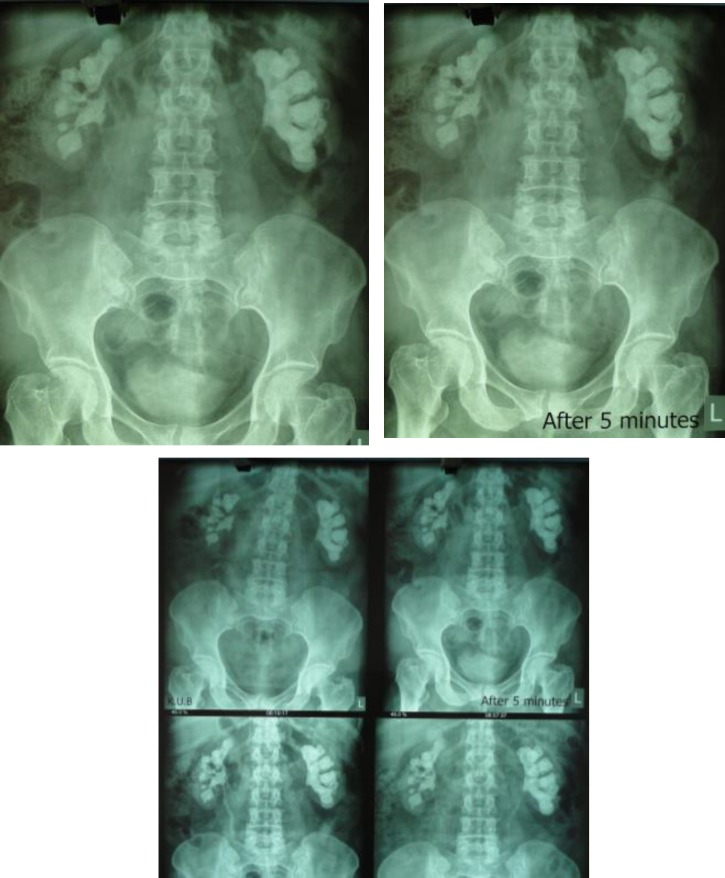


## Discussion

Staghorn calculi are a major threat to health due to recurrent urinary tract infections (UTI) (-). In this study, at the first operation, *S.cohnii sub.urealyticum* was removed only from the stone culture. After 7 years this bacterium was isolated from both cultures (urine and stone). It seems this species can cause the formation of kidney stone.

This bacterium has been reported very low in different databases (3). As in Koksal study, only one *S.cohnii sub.urealyticum* was identified among 200 CNS isolates from blood samples (8). Also, Ferreira reported three *S.cohnii sub.urealyticum* were separated from 152 different clinical samples (9). Only one *S.cohnii sub.urealyticum* was isolated from 64 CNS in Marsik’s study (10). 

In our earlier study, one of 3 isolated *Staphylococus *was* S.cohnii sub.urealyticum* among 45 studied kidney stones (11). Identification of this bacterium in clinical samples is not remarkable. It may be due to the difficulty of clinical and paraclinical diagnosis. Recognition of* S.cohnii sub.urealyticum* in stone is not simple because clinical symptoms are not specific in the patients. Besides, this bacterium is considered as an opportunistic pathogen and has not been investigated in medical laboratory. In addition, routine diagnostic tests in clinical laboratory for the detection of this bacterium are not enough (12, 13). 

In conclusion the isolated bacterium in the present case was *S.cohnii sub.urealyticum* which was cultured from the stone but not the urine. However, after several years the same organism was isolated in both stone and urine indicating a possible contribution of this microorganism in the recurrence of staghorn stone.

## References

[1] Piette A, Verschraegen G (2009). Role of coagulase-negative staphylococci in human disease. Vet Microbiol.

[2] Winn WC, Allen SD, Janda WM (2006). Koneman's color atlas and textbook of diagnostic microbiology.

[3] Soldera J, Nedel WL, Cardoso PR, d'Azevedo PA (2013). Bacteremia due to Staphylococcus cohnii ssp. urealyticus caused by infected pressure ulcer: case report and review of the literature. Sao Paulo Med J.

[4] Wein AJ, Kovoussi LR, Novick AC, Parlin AW, Peters CA ( 2012). Campbell-Walsh urology.

[5] Takeuchi H, Yoshida O (1993). Treatment of staghorn calculi on the basis of composition and structure. Hinyokika Kiyo.

[6] Thomas B, Tolley D (2008). Concurrent urinary tract infection and stone disease: pathogenesis, diagnosis and management. Nat Clin Pract Urol.

[7] Vaidyanathan S, Soni B, Biering-Sorensen F (1998). Recurrent bilateral renal calculi in a tetraplegic patient. Spinal Cord.

[8] Koksal F, Yasar H, Samasti M (2009). Antibiotic resistance patterns of coagulase-negative staphylococcus strains isolated from blood cultures of septicemic patients in Turkey. Microbiol Res.

[9] Ferreira RB, Iorio NL, Malvar KL (2003). Coagulase-negative staphylococci: comparison of phenotypic and genotypic oxacillin susceptibility tests and evaluation of the agar screening test by using different concentrations of oxacillin. J Clin Microbiol.

[10] Marsik F, Brake S (1982). Species identification and susceptibility to 17 antibiotics of coagulase-negative staphylococci isolated from clinical specimens. J Clin Microbiol.

[11] Shafi H, Shahandeh Z, Heidari B (2013). Bacteriological study and structural composition of staghorn stones removed by the anatrophic nephrolithotomic procedure. Saudi J Kidney Dis Transpl.

[12] Stefani M, Del Rosso A, Saldutto P, Galatioto G P, Vicentini C (2012). Intrascrotal Abscess, Propionibacterium acnes and Staphylococcus cohnii ssp. cohnii: A Case Report and Review of the Literature. Case Rep Urol.

[13] Forbes BA, Sahm DF, Weissfeld AS (2007). Baily and Scott's diagnostic micbiology.

